# Nanotechnology Driven Cancer Chemoradiation: Exploiting the Full Potential of Radiotherapy with a Unique Combination of Gold Nanoparticles and Bleomycin

**DOI:** 10.3390/pharmaceutics14020233

**Published:** 2022-01-20

**Authors:** Ocean Han, Kyle Bromma, Nicholas Palmerley, Ariadne T. Bido, Mesa Monica, Abdulaziz Alhussan, Perry L. Howard, Alexandre G. Brolo, Wayne Beckham, Abraham S. Alexander, Devika B. Chithrani

**Affiliations:** 1 Physics and Astronomy, University of Victoria, Victoria, BC V8P 5C2, Canada; oceanhan@uvic.ca (O.H.); kbromma@uvic.ca (K.B.); nicholaspalmerley@uvic.ca (N.P.); alhussan@uvic.ca (A.A.); wbeckham@bccancer.bc.ca (W.B.); AAlexander3@bccancer.bc.ca (A.S.A.); 2 Department of Chemistry, University of Victoria, Victoria, BC V8P 5C2, Canada; atuckmantelbido@uvic.ca (A.T.B.); agbrolo@uvic.ca (A.G.B.); 3 Department of Biochemistry and Microbiology, University of Victoria, Victoria, BC V8P 5C2, Canada; monikm@uvic.ca (M.M.); phoward@uvic.ca (P.L.H.); 4 Centre for Advanced Materials and Related Technologies (CAMTEC), University of Victoria, Victoria, BC V8P 5C2, Canada; 5 BC Cancer, Victoria, BC V8R 6V5, Canada; 6 Division of Medical Sciences, University of Victoria, Victoria, BC V8P 5C2, Canada; 7 Department of Computer Science, Mathematics, Physics and Statistics, Okanagan Campus, University of British Columbia, Kelowna, BC V1V 1V7, Canada

**Keywords:** gold nanoparticles, bleomycin, DNA damage, radiotherapy, tumor cells, cell proliferation

## Abstract

One of the major issues in current radiotherapy (RT) is the associated normal tissue toxicity. Enhancement of the RT effect with novel radiosensitizers can address this need. In this study, gold nanoparticles (GNPs) and bleomycin (BLM) were used as a unique combination of radiosensitizers. GNPs offer a two-fold promise as a delivery vehicle for BLM and as a radiosensitizing agent. In this study, GNPs were functionalized and complexed with BLM using a gold-thiol bond (denoted GNP-BLM). Our results show that there was a 40% and 10% decrease in cell growth with GNP-BLM vs. free BLM for the MIA PaCa-2 and PC-3 cell lines, respectively. Testing the GNP-BLM platform with RT showed an 84% and 13% reduction in cell growth in MIA PaCa-2 cells treated with GNP-BLM and GNPs, respectively. Similar results were seen with PC-3 cells. The efficacy of this approach was verified by mapping DNA double-strand breaks (DSBs) as well. Therefore, this proposed incorporation of nanomedicine with RT is promising in achieving a significantly higher therapeutic ratio which is necessary to make a paradigm change to the current clinical approach.

## 1. Introduction

According to current statistics, more than half of cancer patients receive radiotherapy (RT), and, in some cases, RT is the only treatment option [1,2]. Therefore, RT is an integral component of the curative-intent treatment of many tumors. In RT, high doses of radiation are used to kill cancer cells and shrink tumors via direct and indirect mechanisms [1]. One of the major issues in RT is the close proximity of adjacent organs at risk to the treatment volume; thus, significant normal tissue toxicities limit the treatment dose and prevent the dose escalation necessary to enhance local tumor control. Therefore, enhancing the effect of RT has tremendous potential to maximize the dose to the tumor while mitigating damage to surrounding normal tissue [3,4]. Both of these issues are being addressed using nanotechnology [5,6]. For example, high atomic number nanomaterials may have potential as radiosensitizing agents, while hollow nanocubes with high-index facets are being tested to overcome the radiation side effects to normal tissue [5,6]. The focus of this study is to improve the effect of a local RT dose as discussed next.

One of the current strategies to preferentially increase the local RT effect is to add a radiosensitizer to current RT protocols [7,8,9,10,11]. Radiosensitizers can enhance the killing effect on tumor cells by accelerating DNA damage, disrupting DNA repair, and indirectly producing free radicals. Considerable efforts have been made to develop radiosensitizers that are highly effective when combined with RT. Some of the available chemical radiosensitizers are cisplatin, docetaxel, paclitaxel, gemcitabine, capecitabine, and bleomycin. Furthermore, novel drugs such as piperlongumine derivatives are being explored; these drugs could induce the presence of reactive oxygen species and regulate the Keap1-Nrf2 protective pathway with the enhancement of radiation-induced DNA damage, G2/M-phase cell cycle arrest, and apoptosis [12]. In addition to these chemical radiosensitizers, noble metal nanomaterials such as gold, silver, and platinum nanoparticles can act as physical radiosensitizers. Among other nanomaterials systems, gold nanoparticles (GNPs) in particular, have become popular since they have several advantages, including their biocompatibility; straightforward synthesis; and easy surface functionalization by the attachment of ligands that can target cancer cells, and organelles therein, or improve lifetime in the bloodstream [13,14,15]. In this study, we examined a novel approach to enhance RT: a combination of two radiosensitizers, GNPs and bleomycin (BLM) was used to attain a curative RT effect. In this case, GNPs served as a radiosensitizing agent and as a carrier for the other radiosensitizer, BLM.

GNP-mediated radiosensitization has been observed in many studies as summarized in a few of the latest review articles [16,17]. Because gold is a high Z material, GNPs were initially thought to enhance radiosensitization at kilovoltage photon energies, due to the increased photoelectric photon absorption. However, Compton interactions are dominant at clinically relevant megavoltage energies, and there is a large discrepancy between the predicted increase in physical dose expected by Monte Carlo (MC) and experimental results [6,18,19]. This discrepancy suggests that chemical and biological components are involved in the GNP-mediated radiosensitization process. The main mechanisms identified as being involved in the biological response of cells to GNP-mediated radiosensitization are the production of reactive oxygen species; oxidative stress; induction of DNA damage; and cell cycle effects, as summarized in recent review articles [20,21,22]. The use of GNPs seems more promising in comparison to earlier attempts using iodine because gold has a higher atomic number than iodine and appears to be more biocompatible. The biocompatibility of GNPs has been studied extensively and depends mainly on their size, concentration, cell type, and treatment time as summarized in a review article [20]. For example, size is an important factor because very small particles (5 nm or less in diameter) have been found to be highly toxic, and larger particles are relatively nontoxic. For our study, we used GNPs of diameter ~12 nm at nanomolar concentrations. Based on previous publications and the results of our current study, GNPs in this chosen size and concentration range appear biocompatible.

Bleomycin is one of the most potent natural anti-tumor drugs used to treat cancers, and it acted as a secondary radiosensitizer in this study [23,24]. However, BLM’s pulmonary toxicity limits its therapeutic effectiveness [25]. BLM usage could be expanded if its delivery in the body could be more targeted. One of the ways to achieve controlled delivery of such drugs is to use nanoparticles (NPs) as delivery vehicles [26,27,28,29,30,31,32,33,34]. Studies have shown that NP-based drug delivery systems can provide improvements over the free drug. For example, GNPs have been efficiently used to deliver tumor necrosis factor-alpha (TNF-α) to tumor sites and its nontoxicity has been validated in a phase I clinical trial [35]. For those studies, 27 nm GNPs were functionalized with TNF-α and polyethylene glycol (PEG). The localized delivery of TNF-α induced vascular leakage in the tumor, reduced the tumor interstitial fluid pressure (IFP), and enhanced tumor uptake. GNPs have also been tested as a vehicle for targeted paclitaxel delivery to tumors [36]. Another early phase 1 clinical trial involved the use of spherical GNPs (NU-0129) to deliver nucleic acids to bypass the blood–brain barrier and target the BcL2L12 gene present in recurrent glioblastoma [37]. Further, silica NPs coated with gold and PEG (gold nanoshells; AuroLase^®^) are being tested in a phase I clinical trial as agents of photothermal therapy [38]. Based on these studies, translation of GNPs to the clinic is still in progress as described in recent reviews [16,17,39].

In this study, we used GNPs to deliver BLM because they are known to be nontoxic and nonimmunogenic [40,41]. GNPs are biocompatible, have radiosensitizing properties, and can carry other radiosensitizing drugs, making them very attractive for the design of advanced systems for cancer treatment. There are three major benefits of using the unique combination of GNPs and BLM with RT: first, BLM can block the cell cycle in the G2/M phase, where DNA is most sensitive to radiation, while GNPs can produce cell damaging species during RT. This complementary behavior in their mechanisms of action is expected to yield a better therapeutic outcome. Second, BLM can be conjugated easily to the GNP surface for their efficient delivery. Among other radiosensitizing chemotherapeutics, BLM is a drug that can bind to the GNP surface using a gold-thiol bond, making it a very attractive agent to use with GNPs. Finally, the GNP surface can be further modified with tumor-targeting molecules. In this study, we have chosen smaller diameter spherical GNPs to take advantage of the surface-to-volume ratio for loading BLM molecules and targeting molecules. A peptide containing the integrin-binding domain “RGD” was added to the NP surface for targeting integrin receptors, which are overexpressed in most tumor cells [29]. To achieve biocompatibility and stabilize RGD conjugation, PEG molecules were also added to the NP surface. The functionalization of BLM, RGD peptide, and PEG was easily achieved through thiol linkages [42]. One of our main objectives was to deliver BLM efficiently using GNP as a carrier [34]. It has been demonstrated that GNPs can be used as an effective carrier for the delivery of BLM into tumor cells [43]. Moreover, we were able to further reduce the concentration of BLM by 16-fold, compared to free drug, but achieve better therapeutic results by targeting of GNPs to tumor cells. Therefore, we suspect that this GNP-based platform, which can deliver two radiosensitizers in a controllable fashion, could potentially overcome radiation dose limitations and improve curative cancer treatments.

## 2. Materials and Methods

### 2.1. Cells and Culture Conditions

Human pancreatic cancer cell line MIA PaCa-2 (ATCC#: CRL-1420™) and prostate cancer cell line PC-3 (ATCC#: CRL-1435™) were obtained from the American Type Culture Collection. For all experiments, cells were cultured in high glucose Dulbecco’s Modified Eagle’s Medium (DMEM; Gibco, Markham, ON, Canada) supplemented with the following ingredients: 10% fetal bovine serum (FBS; Gibco); 1% penicillin/streptomycin (Gibco); and 4 mM of GlutaMax™ (Gibco). All incubations were at 37 °C and 5% CO_2_ levels.

### 2.2. GNP Synthesis, Modification, and Characterization

Gold nanoparticles (GNPs) 10–15 nm in diameter were synthesized with the citrate reduction method: 1.18 mL of 1% tetra chloroauric acid (HAuCl_4_) was brought to a boil in 28.82 mL of ddH_2_O before adding 1.2 mL of 5% sodium citrate tribasic dihydrate (HOC(COONa)(CH_2_COONa)_2_·2H_2_O). The solution was left to boil for another 5 min before removing from the heat and cooling for at least 15 min. GNPs were PEG-ylated with 2 kDa PEG-thiol at a ratio of 300 PEG:1 GNP, before conjugating with an integrin-binding domain RGD peptide (NH_2_-Cys-Lys-Lys-Lys-Lys-Lys-Lys-Gly-Gly-Arg-Gly-Asp-Met-Phe-Gly-SH) at a ratio of 150 RGD: 1 GNP.

For this study, these GNP_PEG-RGD_ complexes (GNP-RGD) were conjugated with bleomycin sulfate (BLM) at a ratio of 250 BLM: 1 GNP_PEG-RGD_. For live-cell confocal imaging, these GNP complexes were tagged with Cy5-labeled PEG-thiol (PEG-Cy5) at a ratio of 211 PEG-Cy5:1 GNP.

To measure size and concentration, GNP complexes were characterized with ultraviolet-visible (UV–Vis) spectrophotometry (λ 365 Spectrophotometer; Perkin Elmer, Waltham, MA, USA). A particle analyzer (LiteSizer 500; Anton Paar, Graz, Austria) was used for dynamic light scattering (DLS) and zeta-potential measurements, measuring hydrodynamic diameter and surface charge, respectively.

### 2.3. Quantification of GNP Uptake Using Inductively Coulples Surface Plasmon Mass Spectrocopy (ICPMS)

All treatment incubations for both cell lines were at a GNP concentration of 1 nM. Both cell lines were plated to attain a cell density of 250,000 cells/well in 12-well plates. In triplicate, cells were incubated (37 °C, 5% CO_2_) for 24 h with two different treatments of GNP complex: GNP-RGD and GNP-RGD-BLM (250). Cells were rinsed three times with phosphate-buffered saline (PBS) and dissociated from the wells with TrypLE™ Express. A hemocytometer was used to count the cells in each TrypLE suspension and 400 µL of each suspension was saved for gold quantification. Samples were treated with 65% aqua regia (3:1 ratio of HCl: HNO_3_ (VWR)) in a 200 °C mineral oil bath for at least 1 h. Hydrogen peroxide was added to complete digestion of cell and GNP content before diluting down to 2.5% *v*/*v* in deionized water. The gold content of each sample was measured with inductively coupled plasma mass spectrometry (ICP-MS; Agilent 8800 Triple Quadrupole).

The number of gold atoms per GNP can be calculated as follows:

$$\frac{Atoms\;per\;unit\;cell\times GNP\;Vol.\;\left[nm^{3}\right]}{Unit\;cell\;Vol.\;\left[nm^{3}\right]}=\frac{4\times \frac{4\pi r^{3}}{3}}{a^{3}}=\frac{2}{3}\pi {\left(\frac{D}{a}\right)}^{3}$$

where *D* is the core diameter of a *GNP* and *a* = 0.408 nm is the length of a unit cell. Gold synthesized using the citrate reduction method has a face-centered cubic lattice with 4 atoms per unit cell. With the gold content and number of cells known in each sample, the number of *GNPs* per cell was calculated as follows:

$$\frac{GNP}{cell}=\frac{Au\;Conc.\;\left[\frac{g}{\mathrm{mL}}\right]\times Sample\;Vol.\;\left[\mathrm{mL}\right]\times N_{A}\left[\frac{\mathrm{atoms}}{\mathrm{mol}}\right]}{Au\;atomic\;mass\left[\frac{g}{\mathrm{mol}}\right]\times \#\;of\;Cells\times \frac{\;Au\;atoms}{GNP}}$$

where $N_{A}$ is Avogadro’s number.

### 2.4. Darkfield and Hyperspectral Imaging (HSI)

Both cell lines were plated on glass coverslips in 6-well plates and seeded to attain 60–80% confluency after 2 days of growth. Cells were left overnight to adhere before a 24-h incubation in 1 nM of GNP-RGD or GNP-BLM (250. Following treatment, cells were rinsed three times with PBS before fixing with 4% paraformaldehyde (PFA) for 15 min at 37 °C. The coverslips were mounted onto glass slides with Permount™ mounting medium. Slides were viewed under a 60× immersion oil lens with a darkfield microscope and a hyperspectral camera (CytoViva) for HSI.

### 2.5. Live-Cell Confocal Imaging

Cells were plated on 3 cm coverslip-bottom dishes to attain at least 60% confluency after 48 h. Dishes were incubated for 24 h in 1 nM of Cy5-labeled GNP-RGD or GNP-BLM (250). Dishes were stained with NucBlue™ Live ReadyProbes™ Reagent containing Hoechst 33,342 dye 20 min prior to viewing under a confocal laser scanning microscope (Zeiss LSM 980) with a 60× immersion oil objective.

### 2.6. Cell Cycle Analysis

For each cell line, cells were plated in 6-well plates to attain 75% confluency after their corresponding incubation times. Cells were dosed with either 1 nM of GNP-PEG-RGD and GNP-BLM for 24 h. The dosed media in all wells was replaced with fresh media following the 24 h dosing period. For each treatment, cells were harvested at three time points: immediately after dosing (total 24 h incubation); 24 h after dosing (total 48 h incubation); and 48 h after dosing (total 72 h incubation).

Following incubation, cells were dissociated with TrypLE (Gibco), resuspended in fresh media, and transferred to 15 mL falcon tubes. After spinning at 1300 rpm for 5 min at 4 °C, cell pellets were resuspended in 1 mL PBS. Samples were spun down again at 1300 rpm for 5 min at 4 °C and resuspended in 1% PFA (diluted in PBS), added dropwise while gently vortexing. Samples were incubated at 4 °C for 15 min to complete fixation before spinning down at 1300 rpm for 5 min at 4 °C, washing again with 1 mL PBS, and centrifuging at 1300 rpm for 5 min. Samples were resuspended in chilled 70% ethanol, while vortexing, before being stored in the freezer at −20 °C.

To prepare samples for flow cytometry, the defrosted samples were centrifuged at 1300 rpm for 10 min at 20 °C, washed in a 0.5% BSA (bovine serum albumin) in PBS solution, and spun down at 1300 rpm for 5 min. Samples were resuspended in PBTB (PBS, 0.5% BSA, 0.1% Triton-X 100) along with RNaseA at a concentration of 100 µg/mL. Samples were shaken in this mixture at 37 °C for 25 min. Samples were further incubated with 10 µg/mL propidium iodide (excitation: 488 nm; emission: 600 nm) on a shaker at 4 °C for 60 min, before centrifuging at 1300 rpm for 5 min at 20 °C.

### 2.7. Proliferation Assay (Concentration-Dependent)

For each cell line, cells were plated in five 96-well plates to attain confluency after 4 days. Each plate was given a different treatment: GNP-PEG- RGD, GNP-BLM, or free BLM. All GNP-based treatments were given a maximum dose of 5 nM in one column (8 wells) and concentrations were serially diluted by half in each consecutive column down to 2^−8^ times the maximum concentration. For the plate dosed with free BLM, the dilution scheme was the same, but with a maximum starting concentration of 40 µM. After a 24 h dose incubation, the media was changed. Cell viability was measured at two time points for all treatments: 24 h after dosing; and 48 h after dosing. To measure cell viability, wells were incubated in PrestoBlue™, diluted to 10% *v*/*v* in media, for 20 min before measuring fluorescence (excitation: 530 nm, emission: 590 nm) with a CytationOne™ Multi-Reader (Biotek).

### 2.8. Proliferation Radiation Experiment

For each cell line, cells were plated in two 96-well plates to attain confluency after 4 days. On each plate, two columns of cells were dosed with 1 nM of GNP-PEG-RGD and GNP-BLM. Cells were also dosed with free BLM at the equivalent concentrations of the GNP-BLM doses (~250 nM) and two columns of wells were left un-dosed as a control. After a 24 h incubation with the doses, the dosed media was removed from all plates and one plate was given a 2 Gy dose from a clinical 6 MV linear accelerator (Varian Truebeam, Palo Alto, CA, USA), while the other was left unirradiated. Cell viability was measured at five time points for all treatments: immediately after radiation; 1 day after radiation, 2 days after radiation; 4 days after radiation; and 6 days after radiation. To measure cell viability, wells were incubated for 20 min in 10% PrestoBlue™ before measuring fluorescence (excitation: 530 nm, emission: 590 nm) with a CytationOne™ Multi-Reader.

### 2.9. DNA Double-Strand Breaks Assay

For immunofluorescence, primary antibody solutions were made from 1:100 dilutions of γH2AX (Ser139; Millipore Sigma, Burlington, MA, USA) and 53bp1 (Ser1778; Cell Signaling Technology, Topsfield, MA, USA) in 0.5% BSA/0.1% Triton-X/PBS solution. Secondary antibody solutions were made from 1:500 dilutions of donkey anti-Mouse, Alex Fluor 647 (ThermoFisher, Markham, ON, Canada) and donkey anti-Rabbit, Alexa Fluor 488 (ThermoFisher). For both cell lines, cells were plated on glass coverslips in 6-well plates and seeded to attain 50% confluency after two days. Cells were left to adhere overnight before incubating with 1 nM of GNP-PEG-RGD and GNP-BLM for 24 h. Additionally, cells were dosed with the equivalent free BLM concentration for GNP-BLM which was 250 nM. Cells were rinsed with PBS and fixed with 4% PFA (200 µL coverslip) for 10 min at 37 °C, and rinsed two more times with PBS. For blocking, 200 µL of 2% BSA/0.1% Triton-X/PBS solution was added to each coverslip and incubated at room temperature for 20 min. At room temperature, each coverslip was incubated, face down on parafilm, in a mixture of 50 µL of each primary antibody solution for 1 h. Coverslips were rinsed twice with 1 mL PBS and once with 200 µL 0.5% BSA/0.1% Triton-X/PBS solution, before incubating with a mixture of 50 µL of each secondary antibody solution for 45 min, in the dark. At room temperature, coverslips were washed twice in 1 mL PBS for 5 min each and incubated in 200 µL of 4% PFA for 5 min, before soaking in 1 mL PBS with three drops of NucBlue™ Fixed Cell DAPI stain for 15 min. Each coverslip was mounted onto a glass slide using a drop of Prolong™ Glass Antifade Mountant and left in darkness for 24 h. Cells were imaged with a confocal laser scanning microscope (Zeiss LSM 980) using a 60× oil immersion objective.

### 2.10. Polarization Modulation Infrared Reflection Absorption Spectroscopy (PM-IRRAS) Measurements

PM-IRRAS spectra were recorded using an Equinox 55 (Bruker, Billerica, MA, USA) equipped with a photoelastic modulator (PEM-90 with II/ZS50 ZnSe 50 kHz optical head. HINDS Instruments Inc., Hillsboro, OR, USA), and liquid nitrogen-cooled MCT IR detector (Kolmar Technologies, Houston, TX, USA). The IR beam was directed to the spotted samples at a grazing angle and used with an external reflection configuration. The samples are measured in a nitrogen-filled chamber to decrease CO_2_ and H_2_O vapor signals. The acquisition time was 15 min for the sum spectra and 90 min for the difference spectra. The spectra were recorded in the range 1000 to 1760 wavenumbers. The final spectra were obtained by dividing the difference spectrum by the sum spectrum for each sample. Next, baseline correction was performed in MATLAB^®^ using the Savitsky-Golay signal removal method, described elsewhere [44].

Four 100 nm gold-coated slides were cleaned with piranha solution (3:1 *v*/*v* sulfuric acid: 30% hydrogen peroxide) for 15 min, then rinsed with deionized water in abundance and dried under N_2_ flow to dissolve any organic contaminant that may be present in the slides.

To the first slide, 50 μL of gold nanoparticles were spotted on its center and left to dry covered from light. This process was carried out for the second and third slide, with gold nanoparticles modified with bleomycin and bleomycin solution in water, respectively. The fourth slide was kept without any particle immobilization for a background check of the slide signal.

### 2.11. Statistical Analysis

For statistical analysis, a one-way analysis of variance (ANOVA) was used. A *p*-value < 0.05 was considered statistically significant. Experiments were repeated three times and the data presented are the average, for all experiments.

## 3. Results and Discussion

### 3.1. Characterisation of Nanoparticle Complexes

**Figure 1:**
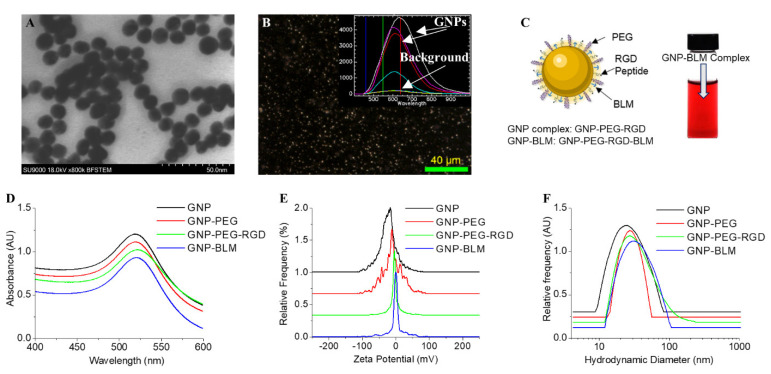
Characterization of NP complexes. (**A**) TEM image of as-made GNPs. (**B**) Darkfield image of GNPs where bright spots are GNPs. Inset figure shows the spectra collected from few of those bright spots using hyperspectral imaging feature. (**C**) Schematic showing the molecules used for functionalization of GNPs (left) and a vial containing the final GNP-BLM complex (right). (**D**–**F**) UV visible absorption, zeta potential, and hydrodynamic diameter of GNP (as-made), GNP-PEG, GNP-PEG-RGD, GNP-BLM, respectively. There is a y offset for D–F for clarity.

Among the size range from 1 to 100 nm, we chose GNPs of diameter ~12 nm for the study. Transmission electron microscopy (TEM) images of GNPs are shown in Figure 1A and the core diameter is 11.3 nm ± 2.1 nm based on measurements of over 100 NPs. As shown in Figure 1B, GNPs were imaged using darkfield and hyperspectral microscopy. The bright specs in the darkfield image are from GNPs, which were further verified by taking a spectral map across the same area using the hyperspectral feature of the same microscope. The spectra collected from a few bright specs are shown in the inset Figure 1B and these spectra match with the reflective spectra of GNPs. We used this technique to map the intracellular distribution of GNPs as well. For this study, GNPs were PEGylated, followed by conjugation of a peptide containing the integrin-binding domain, RGD (see Figure 1C). We used PEG as a stabilizing agent for the conjugation of the RGD peptide. Furthermore, PEG molecules have also been shown to improve the residence time of NPs in the body, which would enable us to exploit the enhanced permeation and retention (EPR) effects present in the tumor microenvironment to accumulate more NPs within the tumor [45]. Therefore, having PEG molecules can be critical in the translation of this in vitro study into an in vivo study in the near future. Since most tumor cells overexpress the RGD domain, the RGD peptide was used to target GNPs into tumor cells efficiently [46]. We exploited the higher surface-to-volume ratio of these smaller NPs for loading targeting molecules and drug molecules onto the surface. The higher surface curvature of smaller NPs also facilitates better receptor–ligand interaction at the cell surface for their efficient intracellular delivery [47].

Functionalized GNPs were characterized at each step during the conjugation process using ultraviolet (UV) absorption spectrometry, zeta potential, and dynamic light scattering (DLS) as shown in Figure 1D–F, respectively. We summarized these results in a table in Supplementary Section S1. The UV–Vis spectrum of citrate-capped GNPs had a peak of 517 nm which corresponds to approximately ~11 nm in core diameter. Data gathered from TEM images (Figure 1A) further support UV data in Figure 1D. The GNP-PEG complex was assembled through sequential conjugation of PEG and RGD at optimized ratios of 300:150 per GNP. The peak red shifted to 519 nm after conjugation with RGD peptide and PEG. The GNP-PEG-RGD complex will be referred to as the GNP complex from now onwards for simplicity. Stabilized GNP complex was used to conjugate BLM and the final complex is referred to as GNP-BLM. In order to prepare the GNP-BLM complex, 250 BLM molecules were added for each GNP. Based on UV and DLS data, adding PEG of molecular weight of 2000 Da led to a slight increase in the diameter of as-made GNPs. However, there was no significant change in size with the addition of RGD peptide and BLM since their sizes were comparable to the size of PEG molecules: 1760 Da for RGD peptide and 1512 Da for BLM. Based on zeta potential data, there was a reduction in the overall negative charge of NPs with the addition of PEG, RGD peptide, and BLM molecules (Figure 1F). This could be due to the removal of negatively charged citrate molecules by neutral PEG molecules and positively charged RGD peptide molecules and BLMs. The complexes were stable since UV, DLS, and Zeta measurements remained the same up to 48 h post conjugation. A vial containing the GNP-BLM complex is included in Figure 1C and the color of the solution shows no sign of apparent NP aggregation. From here on in, GNP complex and GNP-BLM complex are abbreviated terms for GNP-PEG-RGD and GNP-PEG-RGD-BLM, respectively. Our next goal was to test the action of the GNP-BLM complex as compared to the free drug.

### 3.2. Action of GNP-BLM Complex vs. Free Drug

A large majority of commonly used radiosensitizing drugs are small molecules that are administered systemically (orally or intravenously). Such drugs suffer from one major limitation: only a very small percentage of the drug arrives at the tumor site while the rest of it distributes throughout the rest of the body, often inducing toxic side effects in healthy tissue. One of the ways to overcome this challenge is to facilitate the controlled delivery of drugs. For example, the use of NPs has been shown to reduce the required concentration of the drugs significantly due to their controlled delivery [35,48]. In this study, we use GNPs as a carrier for BLM. Due to its large polar surface area, BLM is unable to cross the cell membrane by free diffusion. Studies indicate that the positively charged tail of bleomycin might be key to cellular uptake when the free drug is used for therapeutics [49]. Therefore, placing them on an NP surface using a gold-thiol bond is expected to improve the delivery of BLM [43].

**Figure 2:**
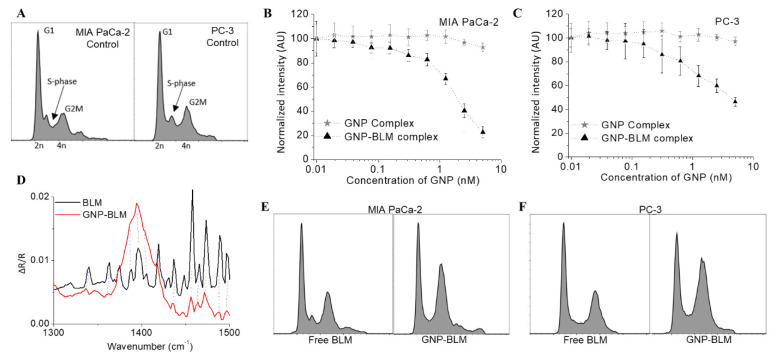
Action of GNP-BLM complex. (**A**) The phases of the cell cycle. As a cell prepares for division it goes through three different phases: G1 is the gap between M and S phase, DNA replication occurs in S phase and G2 is when the cell prepares for mitosis (**B**,**C**) Cell proliferation as a function of concentration for GNP-BLM and GNP complexes IN MIA PaCa-2 and PC-3, respectively. *Y*-axis represents cell viability. (**D**) Polarization modulation infrared reflection absorption spectroscopy data showing the presence of BLM on GNPs. (**E**,**F**) Distribution of cell cycle phase after treatment with 1 nM concentration of GNP complex, GNP-BLM, and free BLM in MIA PaCa-2 and PC-3.

Most anti-tumor drugs act by modulating the cell cycle of the tumor cell population. The cell cycle plays a significant role in optimizing the outcome of cancer therapeutics and it can be divided into four major phases; G1, S, G2, and M (see Figure 2A). The genetic information is duplicated during the synthesis (S) phase and the cell divides into two daughter cells during mitosis (M). S phase and mitosis are separated by gap phases, G1 and G2. Progress through the cell cycle is regulated by several checkpoints, which monitor the state of the cell and its extracellular environment as it prepares for division. It is known that blocking the cell cycle at G2/M can enhance the radiation sensitivity, as the cells’ DNA is most vulnerable to radiation in this phase [50]. Therefore, one of our goals was to use a radiosensitizing drug such as BLM to block the cell cycle in the G2/M phase. In this study, we examine the dual role of GNPs as a radiosensitizer and as a carrier for BLM.

We first investigated the variation of cell viability as a function of the concentration of the GNP-BLM complex as illustrated in Figure 2B for both cell lines. GNP complex (with no BLM) was biocompatible since it did not add any toxicity or cell cycle changes within the concertation range considered for this study (see Figure 2B,C). Based on previously published data and our data, the IC-50 values of free BLM for MIA PaCa-2 and PC-3 are in the micromolar range. By complexing BLM with GNPs, it is possible to improve delivery to cells, requiring smaller BLM concentrations to reach corresponding IC-50 values (Figure 2C). The significant advantage here is the ability to deliver the drug in a controllable fashion since GNPs can be targeted to tumors in vivo [19,45,51]. The current major problem with BLM is its normal tissue toxicity once administered as a free drug. According to previously published work, a gold concentration of 10 µg/mL (~1 nM for GNPs of this size) can be achieved in vivo and it is sufficient to cause enough radiosensitization at clinically relevant MeV energies [19,45,51]. Based on these findings, we chose a 1 nM concentration of GNP complexes for our cellular uptake studies since NPs will be used as a vehicle for the delivery of the drug. The plots in Figure 2B also indicate that the GNP complex (without the drug) did not affect the viability of cells even at a 5-fold higher concentration (5 nM) than the one used (1 nM) for this study. We also evaluated the change in the phase of the cell populations after 24 h of incubation with these GNP-BLM complexes and free drug (see Figure 2C).

Polarization modulation infrared reflection absorption spectroscopy (PM-IRRAS) spectra of solid BLM compared with GNPs modified with BLM show that BLM is present in the GNPs after modification. The dashed lines in Figure 2D indicate that several BLM characteristic peaks are seen in the GNP-BLM complex in the region between 1300 and 1500 wavenumbers. These peaks are related to BLM and are not present in either the non-modified GNP or the bare substrate slide. Due to the complexity of many different molecules present on the GNP surface, it is not very clear that BLM is on the GNP surface according to the spectra in Figure 2D. This is one of the reasons we used other characterization techniques (see Figure 1) to show indirect evidence of BLM on GNPs.

Furthermore, we tested the effect of GNP-BLM at 1 nM concentration vs. free drug on cell cycle as shown in Figure 2E for MIA-PaCa-2 cells (see Supplementary Section S1.3 for data corresponding to PC-3 cells). As expected, we noticed that the GNP-BLM complexes had a relatively higher proportion of cells in the G2/M phase compared to cells treated with the equivalent concentration of the free drug after 24 h. This result suggests that GNPs improved BLM delivery into these tumor cells and shows the benefit of using NPs as a carrier for the drug. Thus, this type of platform may improve delivery and minimize associated drug normal toxicities.

The effect of NPs themselves on the cell cycle has been studied by several groups [52,53,54]. NPs have been shown to cause cell cycle arrest, including G_2_/M and G_0_/G_1_ arrest. However, the type and extent of cell cycle arrest vary depending on the composition, size, size distribution, surface modification, and subsequent surface derivatization of NPs [52,53]. A recent study has shown that GNPs of diameter 20 nm did not cause any variation in the cell cycle [54]. In our study, we used 12 nm diameter GNPs coated with PEG and RGD peptide molecules (GNP complex) for complexing with BLM molecules. However, the size, surface functionality, and concentration of the GNP complex by itself did not alter cell cycle in our study as illustrated in the second panel in Figure 2E. The data corresponding to PC-3 cells are given in Supplementary Section S1.3.

### 3.3. Intracellular Uptake and Distribution of GNP-BLM and GNP Complexes

**Figure 3:**
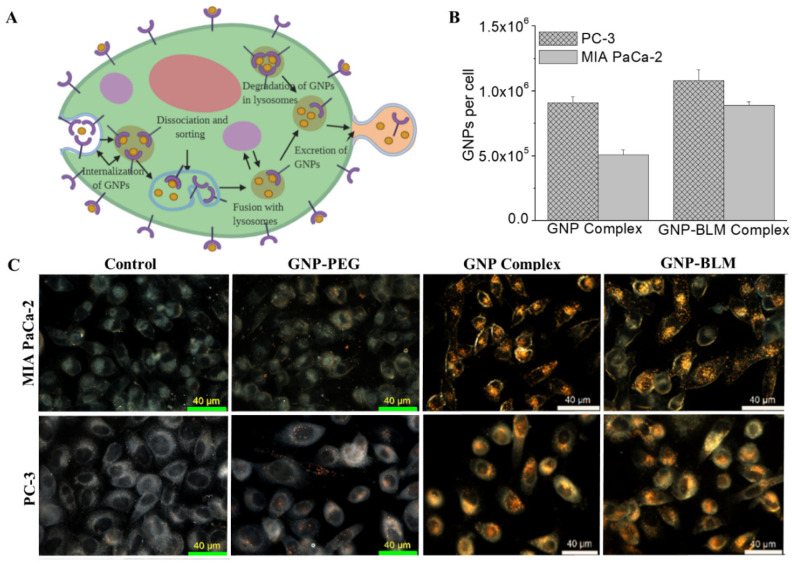
Cellular uptake of GNP complexes. (**A**) endo-lysosomal path of NPs within a cell. (**B**) Cellular uptake comparison of GNP-BLM and GNP. (**C**) Darkfield (scale bar = 40 μm) imaging of control cells and cells internalized with GNPs, GNP–PEG, GNP complex (GNP-PEG-RGD) and GNP-BLM (GNP-PEG-RGD-BLM). Yellow bright spots in darkfield images indicates the presence of NP clusters.

**Figure 4:**
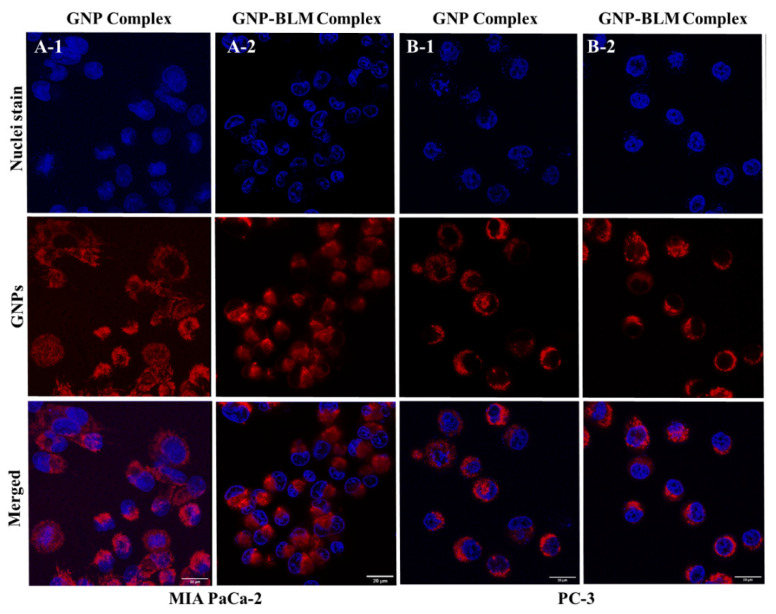
Qualitative analysis of cellular uptake of GNP complexes using confocal imaging. (**A**) NPs localized within MIA PaCa-2 cells. (**A-1**) GNP complex; (**A-2**) GNP-BLM complex. (**B**) NPs localized within PC-3 cells. (**B-1**) GNP complex; (**B-2**) GNP-BLM complex. GNPs are labeled in red and nuclei are labeled in blue. (Scale bar = 20 μm).

We studied the extent of cellular uptake of GNP-BLM complexes as illustrated in Figure 3. We believe that these GNP-BLM complexes are internalized via an endocytosis process (Figure 3A). In our study, GNP-BLM complexes are functionalized with a peptide containing the integrin-binding domain, RGD, to promote receptor–ligand interactions at the cell surface during endocytosis. Upon entering the cells, GNPs are trafficked through the cell via the endo-lysosomal pathway, as illustrated by the schematic in Figure 3A. To fully understand the internalization of the GNP-BLM complex, both quantitative and qualitative techniques were employed as illustrated in Figure 3B,C and Figure 4, respectively. Additional images are provided in Supplementary Section S2. The concentration of GNP-BLM complex was 1 nM since such concentrations are feasible in vivo and also in a clinical setting. We also compared the uptake of GNP-BLM complex to GNP complex (GNP-PEG-RGD used for BLM conjugation).

To quantify the amount of GNPs present within cells, inductively coupled plasma mass spectroscopy (ICP-MS) was used (see Figure 3B) and we observed a slight increase in GNP uptake for the GNP-BLM complex. We believe that this could be due to the slightly less negative surface charge of the GNP-BLM complex (see Figure 1E and Supplementary Section S1). The reduction in negative charge in the GNP-BLM complex could be due to further replacement of negatively charged citrate surfactant with BLMs. According to a theoretical model put forward by Cho et. al., NP uptake process occurs in two steps: adsorption onto the cell membrane of the cell and internalization by the cell [55]. Our results are consistent with recent experimental and theoretical studies that showed uptake of NPs increased when their zeta potential was less negative, due to the negative charge of the cell membrane surface [55,56]. NP size also plays a significant role in their cellular uptake and transport process based on previous experimental and theoretical studies [56,57]. However, there were no significant differences in the overall size of the three GNP complexes based on characterization data in Figure 1. The sizes of PEG molecules (~2000 Da), RGD peptide (~1760 Da), and BLMs (~1520 Da) added to GNP surface are comparable to each other, explaining why additional conjugation of RGD and BLM did not significantly change the size. Qualitative analysis of GNP-BLM and GNP complexes was carried out to map NP distribution within cells, and we used darkfield and confocal microscopy as shown in Figure 3C and Figure 4. Both imaging techniques clearly showed GNP presence within cells and our imaging data are consistent with our quantitative data in Figure 3B. Our uptake studies demonstrated that targeting of NPs into cells was not reduced after drug conjugation; in fact, there was a slight advantage due to the less negative nature of the final GNP-BLM complex. It is also not yet known how the presence of BLM affects the excretion of GNPs. Previous studies have shown size, surface properties of NPs, and the cell cycle affects their excretion process [50,58]. Therefore, one of our future studies includes studying the effect of BLM on the GNP excretion process both in vitro and in vivo. We will next discuss the extent of GNP-mediated delivery of BLM.

### 3.4. Evaluation of GNP Mediated Drug Delivery

**Figure 5:**
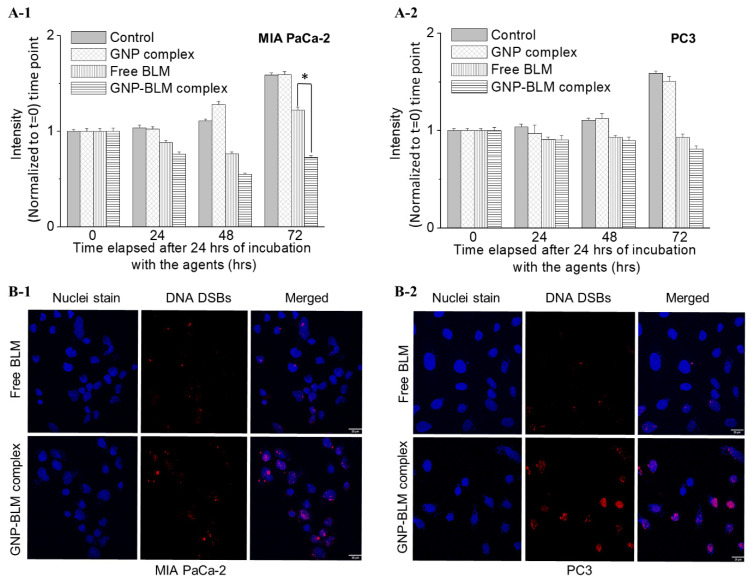
GNP-mediated drug delivery. (**A-1**,**A-2**) Progression in cell growth in MIA PaCa-2 and PC-3 after treatment with GNP-BLM, GNPs, and free BLM for a 24 h time period, respectively. * indicates *p* < 0.05. (**B-1**,**B-2**) Mapping the DNA DSBs after 24 h of the treatment for MIA PaCa-2 (left panel) and PC-3 (right panel), (scale bar = 20 μm).

The main mode of action of BLMs is thought to be related to their ability to mediate both single-stranded breaks and double-stranded breaks (DSBs) [49]. In our study, we are probing DNA DSBs since it is the more lethal one. Based on the results of previous in vitro studies, the DNA-cleaving actions of BLM are dependent on oxygen and metal ions. It is believed that bleomycin chelates metal ions (primarily iron) producing a pseudo enzyme that reacts with oxygen to produce superoxide and hydroxide free radicals that cleave DNA. In order to evaluate the effect of GNP-mediated drug delivery, we probed both cell proliferation as well as DNA damage. To measure the effect on cell proliferation due to the treatment, cells were introduced with fresh media after treatment with the agents for 24 h. Cell proliferation was monitored for a 72 h time period and the results are shown in Figure 5A-1,A-2. There was no effect of GNPs on the proliferation of cells and it clearly shows that the GNP complex did not cause any added toxicity to cells. There was a decrease in cell proliferation or growth with the treatment of the GNP-BLM complex in contrast to free BLM at the 72 h time point. For example, there was a 40% and 10% decrease in cell growth with the treatment of GNP-BLM vs. free BLM at the 72 h time point for MIA PaCa-2 and PC-3, respectively. The effect of BLM on the cell cycle phase of tumor cell populations over time was also evaluated (see Supplementary Section S3). More cells were synchronized in the G2/M phase when treated with GNP-BLM vs. free BLM. These results indicate better delivery of BLM using GNPs. Based on the data presented in Figure 5A-1,A-2, the MIA PaCa-2 cell line seems to have a better response to the treatment compared to PC-3. The difference in treatment response could be a combined effect of MIA PaCa-2 having a longer doubling time and a lower IC-50 than PC-3. For example, cell doubling times for PC-3 and MIA PaCa-2 are ~28 and 40 h, respectively and their IC-50 values are 0.2 and 11 µM, respectively.

Our second approach to measure the efficacy of the drug delivery was to probe the number of DNA double-strand breaks (DSBs). When there is DNA damage, DNA repair proteins such as 53BP1 and γH2AX localize at those sites of DNA damage. Therefore, we probed those two repair proteins to measure the DNA damage [6]. We have presented data for γH2AX repair protein in Figure 5B-1,B-2 since there is significant co-localization of foci corresponding to 53BP1 and γH2AX (see Supplementary Section S4). There is an enhancement in DNA DSBs in nuclei of cells treated with GNP-BLM in contrast to the ones treated with free drug (Figure 5B-1,B-2).

Based on the data from proliferation and DNA DSBs assays, we can conclude that GNP-mediated BLM delivery is more efficient than the free drug itself. We also noticed significant morphological damage to the nucleus of treated with GNP-BLM vs. free BLM (Supplementary Section S5). A previous study has demonstrated GNP-mediated BLM delivery, albeit with a higher GNP to BLM ratio of 1:4050 [43]. In our study, we reduced the ratio ~16-fold to 1:250, but still achieved similar results by improving the targeting of GNP complexes using a peptide containing the integrin-binding domain, RGD. Other efforts have been made to improve BLM delivery using liposomal carriers [59]. However, there was no significant advantage at in vitro level due to very high IC-50 values. In our study, we were able to reduce IC-50 by a significant margin by targeted delivery using GNPs. Another advantage we have is that GNPs not only act as a carrier of this radiosensitizing drug, BLM, but also it can act as a radiosensitizing agent. As explained next, we exploited this dual role of GNPs as a radiosensitizer and a drug carrier to further improve the outcome of cancer radiotherapy.

### 3.5. Combined Approach of GNPs, BLMs, and Radiation

**Figure 6:**
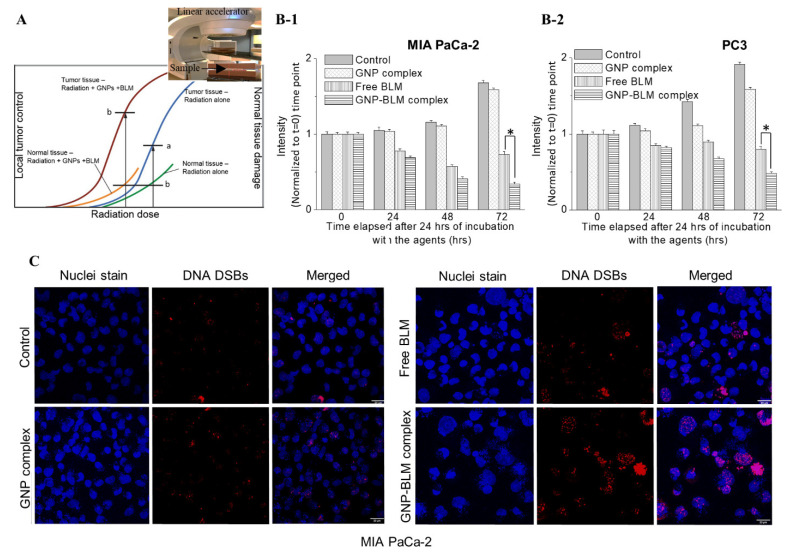
Cancer chemoradiation. (**A**) Addition of radiosensitizers to tumor allows shifting the tumor controllability curve to the left. This would enable lowering of RT dose while minimizing normal tissue toxicity. (**B**) Progression in cell growth in MIA PaCa-2 (**B-1**) and PC-3 (**B-2**) after a 2 Gy RT dose from a clinically used linear accelerator (inset of A). * indicates *p* < 0.05. (**C**) Mapping the DNA DSBs after 24 h of the RT treatment for MIA PaCa-2. The scale bars are 20 µm.

Radiotherapy is an essential element of curative treatment of many cancers. The major limitation to reaching a curative RT dose in high-risk (locally advanced) non-metastatic tumors is the high sensitivity to radiation and subsequent damage to the surrounding normal tissues as illustrated in Figure 6A. Currently, we are at the limit of radiotherapy doses given to patients, creating a clear need for novel methods to enhance it and further improve survival. Therefore, enhancing targeted delivery of radiotherapy has tremendous potential to maximize the effect of the dose given to the tumor and minimize the dose given to normal tissue [39,60]. One of the current strategies to preferentially increase tumor radiation dose effect is to add a radiosensitizer to radiotherapy [7,8,9,10,11]. In order to enhance the curative radiotherapy dose, while protecting surrounding normal tissue, we have tested a novel approach where a unique combination of two radiosensitizers, GNPs and BLM was added to radiotherapy. The combination of GNPs and BLM is unique since GNPs can act as a physical radiosensitizer while BLM can block the cell cycle in the G2/M phase, which is the most sensitive phase to radiation. We used a clinically relevant linear accelerator for the treatment of cells, where the inset figure in Figure 6A shows the experimental setup.

A radiation dose of 1.8–2 Gy would be considered in “conventional” fractionated treatment. In particular, fractionation is generally utilized in protocols of concurrent chemoradiotherapy (i.e., 50 Gy in 25 fractions, 60 Gy in 30 fractions, 45 Gy in 25 fractions). In this study, we tested the efficacy of GNP-mediated chemoradiation. Moreover, we used a single RT dose of 2 Gy to mimic one fraction currently used in chemoradiation. Hence, it is important to mention that the effect we see in Figure 6 is only after a single 2 Gy dose of radiation with a clinically used linear accelerator. In addition, we only used a 1 nM GNP concentration since it can be achieved in vivo, based on previous publications. After the radiation dose was delivered, the proliferation of cells was monitored over a 72 h time period. There is a significant reduction in cell growth in the cells treated with GNP-BLM vs. free BLM (see Figure 6B-1,B-2). This clearly shows that GNPs not only act as a radiosensitizer, but also as a platform for carrying another radiosensitizer, BLM. The combined use of GNPs and BLM with RT produced a synergistic therapeutic outcome in contrast to GNP/RT. For example, there was an 84% reduction in cell growth with GNP/BLM with RT vs. 13% for GNP with RT in the MIA PaCa-2 cell line. Similar results were seen with PC-3 for GNP/BLM (75%) and GNP (21%) with RT. We also mapped the extent of DNA DSBs by probing DNA repair proteins, such as γH2AX and 53BP1, as discussed before. Twenty-four hours after the RT treatment, we tracked the presence of γH2AX and 53BP1 proteins using antibodies against them. Foci corresponding to γH2AX and 53BP1 are localized to DSB sites and we have shown only data corresponding to γH2AX in Figure 6C for MIA PaCa-2. The DNA DSBs data for the PC-3 cell line are presented in Supplementary Section S7. Mapping of DNA DSBs after 24 h of radiation was used as a good measure of cell damage in previous studies [6]. Co-localization of these foci was seen in our study, which is consistent with previously published work (see Supplementary Section S4) [6]. As illustrated in Figure 6C, there is an increase in DNA DSBs with the treatment of GNP/BLM with RT vs. GNP or BLM with RT. In addition to the presence of more DNA DSBs, the integrity of the nuclei seemed to be damaged quite significantly when treated with GNP-BLM and RT, evidenced by what appeared to be more micronucleation. Considering that modern fractionated RT plans are applied locally, through the use of IMRT or VMT, we can significantly increase damage within the tumor with GNP-BLM/RT combination. The results shown are quite promising, even with a single fraction of RT from a clinically used linear accelerator.

## 4. Conclusions

Approximately 50% of all cancer patients can benefit from RT in the management of their disease; of these, approximately half present early enough to pursue curative intent [61,62]. Incorporation of GNPs with BLM could quite considerably improve the local RT effect to significantly retard or eradicate tumor growth. We were able to reduce IC-50 of BLM using GNPs as an effective carrier. GNPs are being used as a promising radiosensitizing agent. The cell proliferation and DNA DSBs data demonstrated that the combination of GNP-BLM with RT can significantly enhance cancer cell damage compared to using RT alone or using RT with GNP complex without BLM. Remarkably, this milestone was achieved with a 1 nM concentration of GNPs. Such concentrations can be applied safely in vivo based on previous publications. Our proposed approach has two main advantages: (1) the use of GNPs allows for the controlled delivery of anticancer drugs, which minimizes their side effects; and (2) the proposed approach allows us to reduce the current RT dose used in the clinic, since the presence of two radiosensitizers such as GNPs and BLM within the tumor can enhance the effect of RT significantly based on our experimental findings. Our next goal is to perform an in vivo study to accelerate the use of this unique combination of GNPs, BLM, and RT in a clinical setting. We will first perform a biodistribution study to find the optimum time point for the delivery of the RT dose. Finally, with this enhanced RT effect a reduction in prescription dose is conceivable and with improved (targeted) delivery of anticancer drugs, both could eventually lead to reduced normal tissue toxicities which would result in improving disease control and quality of life for cancer patients. Therefore, this GNP-mediated therapeutic platform will potentially overcome current limitations in RT and pave the way for a new era in curative cancer treatments.

## Data Availability

Datasets generated and/or analyzed during the current study are available from the corresponding author on reasonable request.

## Supplementary Materials

The following are available online at https://www.mdpi.com/article/10.3390/pharmaceutics14020233/s1, Figure S1.1: Nanoparticles were characterized using UV Visible absorption spectroscopy, dynamic light scattering, and zeta potential measurements. The table below summarizes the data for as-made GNP, GNP-PEG, GNP-PEG-RGD (GNP complex) and GNP-BLM complex, Figure S1.2: Polarization modulation infrared reflection absorption spectroscopy data for substrate, free BLM, GNP complex, and GNP-BLM, Figure S1.3: Comparison of cell cycle for control cells, cell treated with GNP complex (GNP-PEG-RGD), Free BLM and GNP-BLM for PC-3 cells., Figure S2.1: Darkfield imaging of GNPs. (A-1) Darkfield image (DFI): Yellow bright specs in the image indicates the presence of NP clusters. (A-2) Hyperspectral image (HSI): Spectral map of NPs. (A-3) A collection of reflectance spectra collected from few GNPs in A-2, Figure S2.2: Darkfield imaging of NPs localized within MIA PaCa-2 cells. (A-1, B-1) Darkfield images (DFI): Yellow bright specs in the image indicates the presence of NP clusters. (A-2, B-2) Hyperspectral images (HSI): Spectral map of NPs. (A-3, B-3) A collection of reflectance spectra collected from few nanoclusters in A-2 and B-2., Figure S2.3: Darkfield imaging of NPs localized within PC-3 cells. (A-1, B-1) Darkfield images (DFI): Yellow bright specs in the image indicates the presence of NP clusters. (A-2, B-2) Hyperspectral images (HSI): Spectral map of NPs. (A-3, B-3) A collection of reflectance spectra collected from few nanoclusters in A-2 and B-2, Figure S3: Distribution of cell cycle phase after treatment with 1 nM concentration of GNP-BLM vs 250 nM free BLM in MIA PaCa-2 and PC-3. (A, B) Dynamics of cell cycle phase distribution for MIA PaCa-2 and PC-3 cells after 24 h treatment with GNP-BLM and free BLM, respectively, Figure S4.1: Imaging of DNA DSBs through mapping of two DNA DSBs repair proteins: 53BP1 and γH2AX. (A-1, A-2) 53BP1 and γH2AX foci for cells treated with free BLM, respectively. (B-1, B-2) 53BP1 and γH2AX foci for cells treated with GNP-BLM, respectively. Scale bar is 20 µm, Figure S4.2: Imaging of DNA DSBs through mapping of two DNA DSBs repair proteins: 53BP1 and γH2AX. (A-1, A-2) 53BP1 and γH2AX foci for cells treated with free BLM, respectively. (B-1, B-2) 53BP1 and γH2AX foci for cells treated with GNP-BLM, respectively. Scale bar is 20 µm, Figure S5: Comparison of the damage to the nucleus with GNP-mediated BLM delivery vs. free BLM. (A-1, A-2) Nuclei of MIA PaCa-2 treated with GNP-BLM vs. free BLM, respectively. (B-1, B-2) Nuclei of PC-3 cells treated with GNP-BLM vs. free BLM, respectively. Scale bar is 20 µm, Figure S6: Comparison of the damage to the nucleus with radiation for control cells, cells treated with GNP complex, free BLM, and GNP-BLM for MIA PaCa-2 and PC-3. Scale bar is 20 µm, Figure S7: Detailed comparison of the damage to the nucleus with radiation for control cells, cells treated with GNP complex, free BLM, and GNP-BLM for PC-3. Scale bar is 20 µm.
