# Supplementary material for: Nanotechnology Driven Cancer Chemoradiation: Exploiting the Full Potential of Radiotherapy with a Unique Combination of Gold Nanoparticles and Bleomycin
Source: Pharmaceutics. 2022 Jan 20;14(2):233. doi: 10.3390/pharmaceutics14020233 (PMC8875790; doi:10.3390/pharmaceutics14020233)
Supplement: Supplementary file 1 [file pharmaceutics-14-00233-s001.zip › pharmaceutics-1499799-supplementary.pdf]

pharmaceutics

MDPI

Supplementary Materials: Nanotechnology driven cancer chemoradiation: Exploiting the full potential of radiotherapy with a unique combination of gold nanoparticles and bleomycin

Ocean Han, Kyle Bromma, Nicholas Palmerley, Ariadne T. Bido, Mesa Monica, Abdulaziz Alhussan, Perry L. Howard, Alexandre G. Brolo, Wayne Beckham, Abraham S. Alexander and Devika B. Chithrani

Supplement section S1: Summary of GNP characterization data
Supplement section S1.1

	UV peak wavelength (nm)	Hydrodynamic diameter (nm)	Zeta potential (mV)
GNP	517	24.9±12.6	-20.5±1.6
GNP-PEG	519	27.0±8.5	-16.8±0.9
GNP-PEG-RGD (GNP complex)	519	27.0±5.4	-5.2±1.0
GNP-PEG-RGD-BLM (GNP-BLM complex)	519	29.3±5.7	-0.8±0.6

Figure S1.1. Nanoparticles were characterized using UV Visible absorption spectroscopy, dynamic light scattering, and zeta potential measurements. The table below summarizes the data for as-made GNP, GNP-PEG, GNP-PEG-RGD (GNP complex) and GNP-BLM complex.

Supplement section S1.2

Polarization modulation infrared reflection absorption spectroscopy data

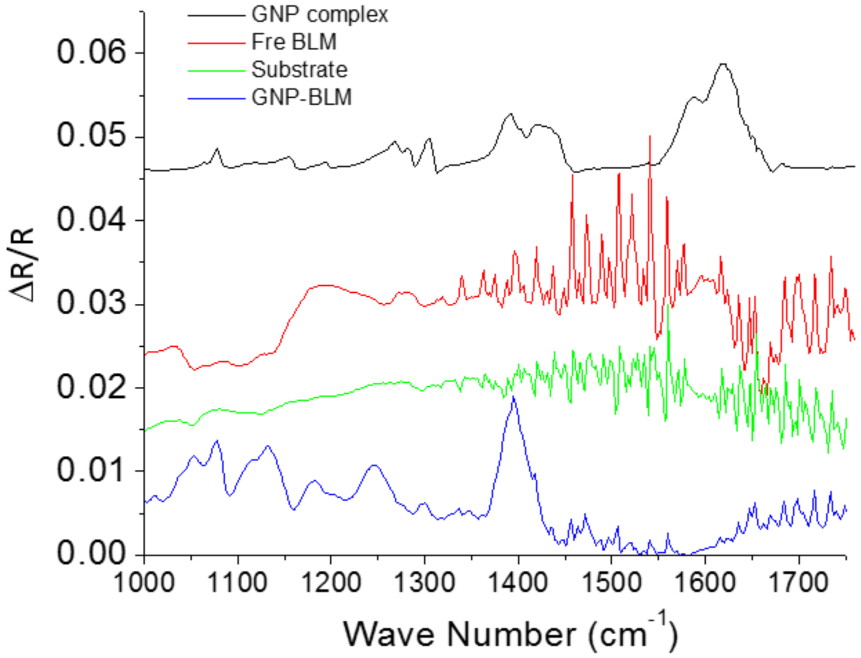

Figure S1.2. Polarization modulation infrared reflection absorption spectroscopy data for substrate, free BLM, GNP complex, and GNP-BLM.

Supplement section S1.3: Effect of GNP complex, GNP-BLM and free BLM on cell cycle

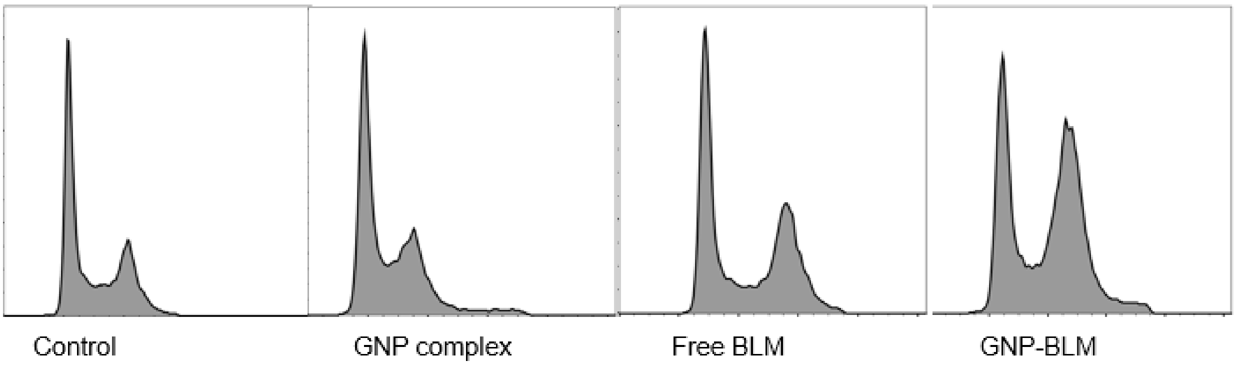

Figure S1.3. Comparison of cell cycle for control cells, cell treated with GNP complex (GNP-PEG-RGD), Free BLM and GNP-BLM for PC-3 cells.

Supplement section S2: Intracellular distribution of GNP-BLM complex and GNP complex. Darkfield and hyperspectral imaging were used to map the NP distribution.

S2.1 Imaging of GNPs using darkfield and hyperspectral feature

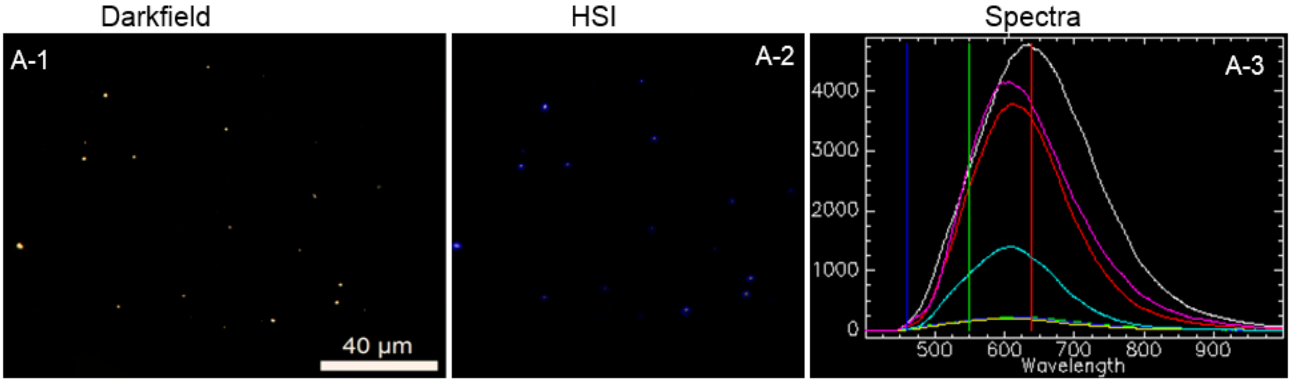

Figure S2.1 Darkfield imaging of GNPs. A-1) Darkfield image (DFI): Yellow bright specs in the image indicates the presence of NP clusters. A-2) Hyperspectral image (HSI): Spectral map of NPs. A-3) A collection of reflectance spectra collected from few GNPs in A-2.

S2.2 Imaging of GNPs in MIA PaCa-2 cells using darkfield and hyperspectral feature

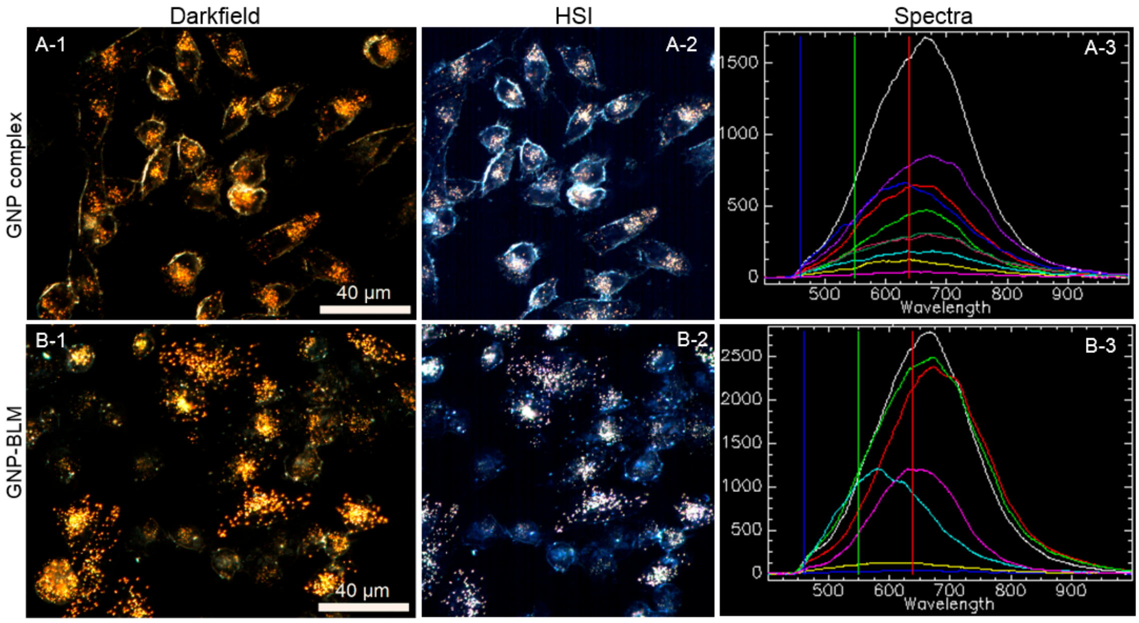

Figure S2.2 Darkfield imaging of NPs localized within MIA PaCa-2 cells. A-1, B-1) Darkfield images (DFI): Yellow bright specs in the image indicates the presence of NP clusters. A-2, B-2) Hyperspectral images (HSI): Spectral map of NPs. A-3, B-3) A collection of reflectance spectra collected from few nanoclusters in A-2 and B-2.

S2.3 Imaging of GNPs in PC-3 cells using darkfield and hyperspectral feature

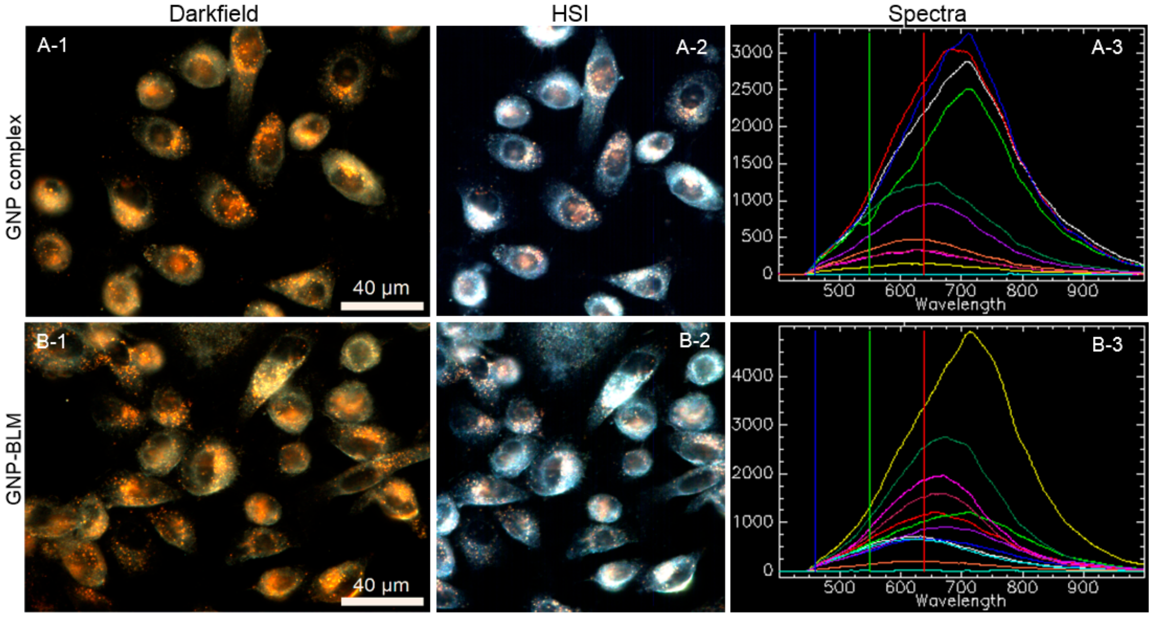

Figure S2.3 Darkfield imaging of NPs localized within PC-3 cells. A-1, B-1) Darkfield images (DFI): Yellow bright specs in the image indicates the presence of NP clusters. A-2, B-2) Hyperspectral images (HSI): Spectral map of NPs. A-3, B-3) A collection of reflectance spectra collected from few nanoclusters in A-2 and B-2.

Supplement section S3: Cell cycle variation as a function of time for cells treated with GNP- BLM vs. free BLM

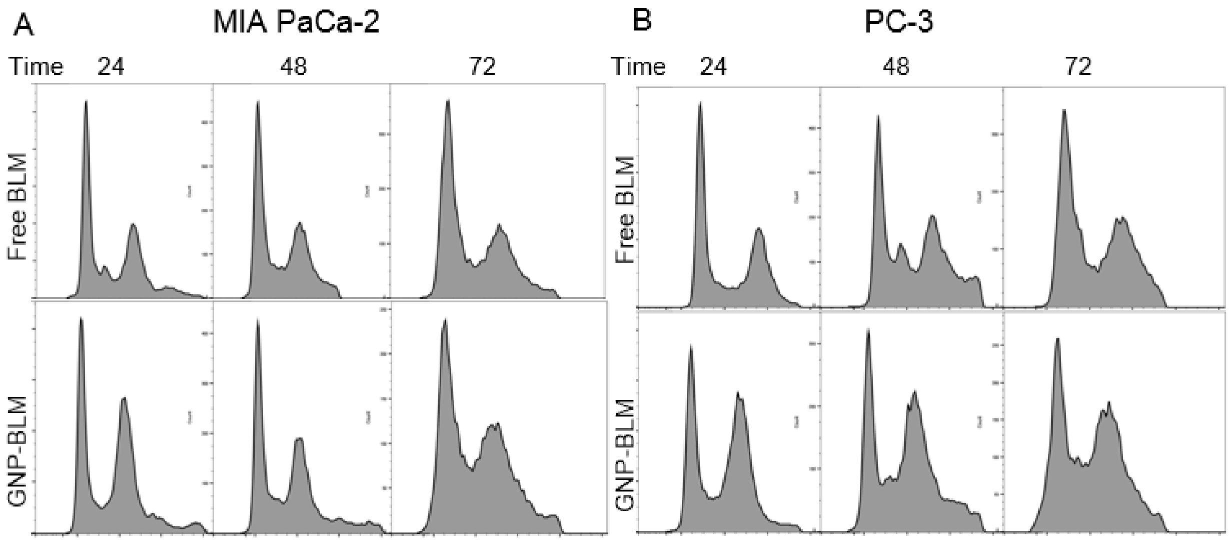

Figure S3. Distribution of cell cycle phase after treatment with 1 nM concentration of GNP-BLM vs 250 nM free BLM in MIA PaCa-2 and PC-3. A, B) Dynamics of cell cycle phase distribution for MIA PaCa-2 and PC-3 cells after 24 hr treatment with GNP-BLM and free BLM, respectively.

Supplement section S4: Imaging of DNA DSBs through tracking of two DNA DSBs repair proteins: γH2AX and 53BP1

S4.1 MIA PaCa-2 cell line

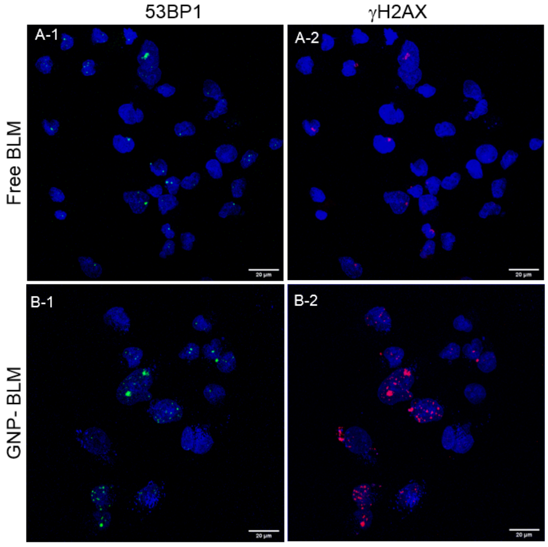

Figure S4.1 Imaging of DNA DSBs through mapping of two DNA DSBs repair proteins: 53BP1 and γH2AX**.** A-1, A-2) 53BP1 and γH2AX foci for cells treated with free BLM, respectively. B-1, B-2) 53BP1 and γH2AX foci for cells treated with GNP-BLM, respectively. Scale bar is 20 μm.

S4.2 PC-3 cell line

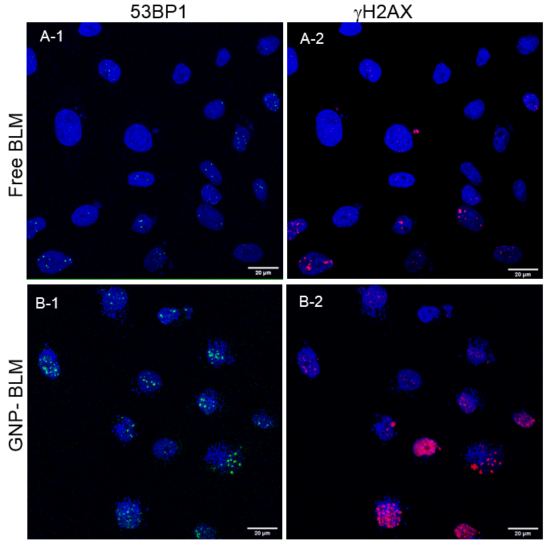

Figure S4.2 Imaging of DNA DSBs through mapping of two DNA DSBs repair proteins: 53BP1 and γH2AX**.** A-1, A-2) 53BP1 and γH2AX foci for cells treated with free BLM, respectively. B-1, B-2) 53BP1 and γH2AX foci for cells treated with GNP-BLM, respectively. Scale bar is 20 μm.

Supplement section S5: GNP-mediated BLM delivery vs. free BLM

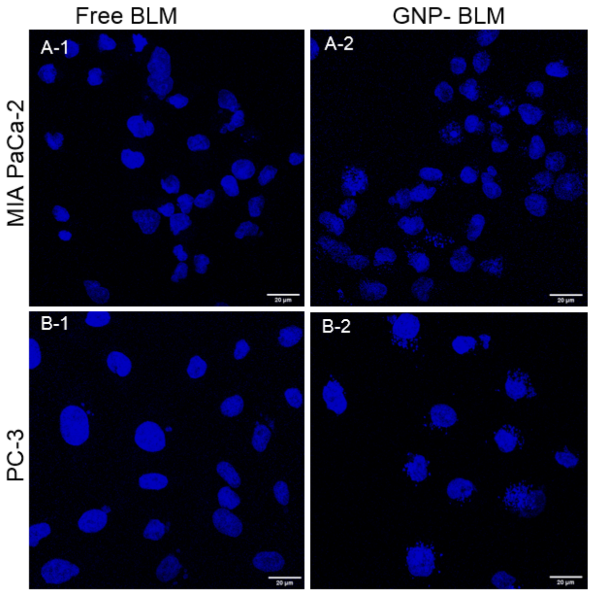

Figure S5. Comparison of the damage to the nucleus with GNP-mediated BLM delivery vs. free BLM. A-1, A-2) Nuclei of MIA PaCa-2 treated with GNP-BLM vs. free BLM, respectively. B-1, B-2) Nuclei of PC-3 cells treated with GNP-BLM vs. free BLM, respectively. Scale bar is 20 μm.

Supplement section S6: GNP-mediated chemoradiation

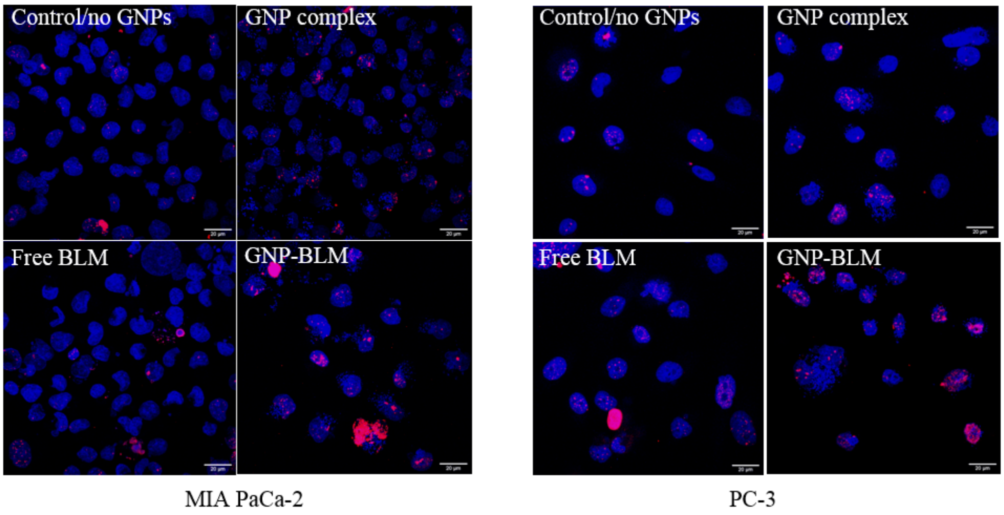

Figure S6. Comparison of the damage to the nucleus with radiation for control cells, cells treated with GNP complex, free BLM, and GNP-BLM for MIA PaCa-2 and PC-3. Scale bar is 20 μm.

Supplement section S7: DNA DSBs data for in PC-3 cells treated with a 2Gy radiation dose with a clinical linear accelerator

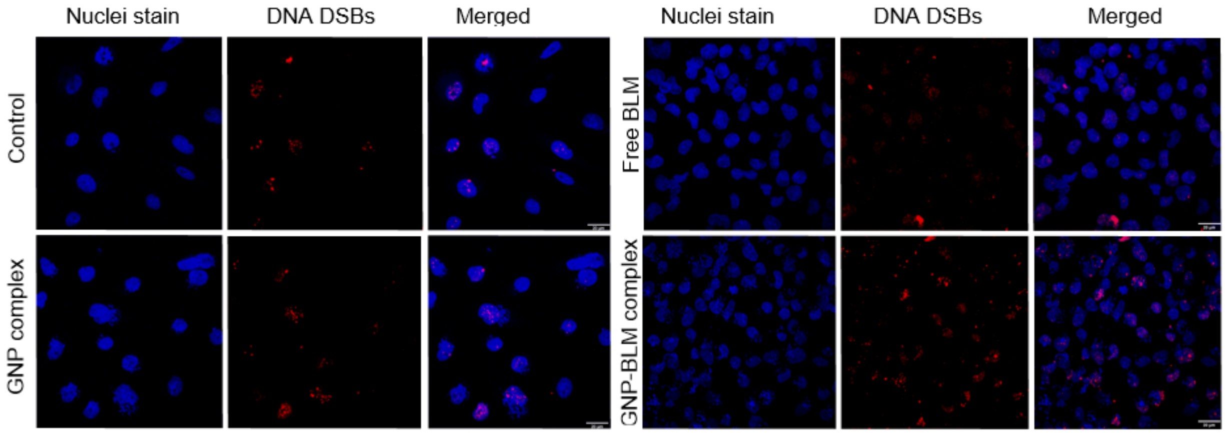

Figure S7. Detailed comparison of the damage to the nucleus with radiation for control cells, cells treated with GNP complex, free BLM, and GNP-BLM for PC-3. Scale bar is 20 μm.
